# Impact of climate change and environmental adversities on maternal and fetal health: the role of clinical practices and providers in mitigating effects and prioritising women's health in the UK

**DOI:** 10.3389/fgwh.2025.1483938

**Published:** 2025-06-13

**Authors:** Athina Samara, Thomas Hanton, Ranee Thakar, Eric Jauniaux, Asma Khalil

**Affiliations:** ^1^ Department of Women’s and Children’s Health, Karolinska Institutet, Stockholm, Sweden; ^2^ Astrid Lindgren Children’s Hospital, Karolinska University Hospital, Stockholm, Sweden; ^3^ FUTURE, Center for Functional Tissue Reconstruction, University of Oslo, Oslo, Norway; ^4^ Fetal Medicine Unit, St George’s University Hospitals NHS Foundation Trust, University of London, London, United Kingdom; ^5^ Royal College of Obstetricians and Gynaecologists, London, United Kingdom; ^6^ Obstetrics and Gynaecology, Croydon University Hospitals NHS Trust, London, United Kingdom; ^7^ EGA Institute for Women's Health, Faculty of Population Health Sciences, University College London, London, United Kingdom; ^8^ Vascular Biology Research Centre, Molecular and Clinical Sciences Research Institute, St George’s University of London, London, United Kingdom; ^9^ Fetal Medicine Unit, Liverpool Women’s Hospital, Liverpool, United Kingdom

**Keywords:** climate change, pregnancy, maternal health, fetal development, environmental pollution, sustainable healthcare, UK healthcare system

## Abstract

The climate crisis poses profound risks to women particularly during pregnancy. With rising global temperatures and increasing frequency of extreme weather events, there is an urgent need for health initiatives and guidelines tailored to the unique vulnerabilities of pregnant individuals. We conducted a review of English-language literature from 2000–2024 using PubMed, Scopus, and Web of Science, focusing on “climate change,” “pregnancy,” and “maternal health,” and included original studies, reviews. Relevant policy documents, including some published in 2025 were also included. We examine the multifaceted challenges posed by climate change, such as extreme weather events, water scarcity, malnutrition, and exposure to environmental pollutants like contaminated air and water, which directly and indirectly affect maternal and fetal health. The review explores the associations between these environmental stressors and adverse pregnancy outcomes, including preterm births, low birth weight, and developmental complications. These challenges are compounded in low-resource settings where healthcare infrastructure is limited, exacerbating inequities in maternal care. Furthermore, we focus on key areas for further investigation, including the long-term health effects of in-utero exposure to pollutants. The review addresses evidence-based strategies to reduce the environmental impact of healthcare through early interventions, innovation, and strengthened initiatives. It emphasises empowering healthcare professionals to educate others, raise awareness among policymakers, advocate for climate-conscious policies, and promote sustainable practices reducing the carbon footprint of the healthcare system, with a focus on the UK. In response to these pressing concerns, leading professional organizations, such as the Royal College of Obstetricians and Gynaecologists (RCOG) in the UK, and the American College of Obstetricians and Gynaecologists (ACOG) in the US, are prioritizing the intersection of climate change and women's health. Their initiatives, which aim to mitigate the climate-change impacts on pregnancies and fetal health by promoting research, raising awareness, and developing actionable strategies, are also highlighted. By amplifying awareness and global collaboration, the suggested strategies aim to protect maternal and fetal health in the face of an escalating climate crisis.

## Introduction

Climate change and environmental pollution collectively pose a significant and escalating threat to human health. The World Health Organization (WHO) recognises these phenomena as among the most critical challenges to future global health, with pollution intensifying the effects of climate change and vice versa (1). The inter-governmental panel on climate change (IPCC) published the Sixth Assessment Report (AR6) in 2023, which identified that 3.3–3.6 billion people are vulnerable to the effects of climate change (2). According to the United Nations climate Promise, climate justice has many dimensions and women are disproportionately impacted by climate change, as they often have less access to resources that would help them adapt to and cope with sudden environmental changes (3). Notably, in a recent article based on discussions within the International Federation of Gynecology and Obstetrics (FIGO) Committee on Climate Change and Toxic Environmental Exposures, committee members emphasize that climate change should be addressed “in the context of women's reproductive health as a public health issue, a social justice issue, a human rights issue, an economic issue, a political issue, and a gender issue, one that demands our attention now for the health and well-being of this and future generations” (4).

Recent literature highlights the risks posed to women's health by various environmental factors, including both human-generated air pollution (5) and the adverse effects of climate change (6). Industrial pollution has degraded air quality and increased environmental contamination from plastics and microplastics (7–9, 12, 25, 145). In addition to the well-established direct impact of extreme climatic events and air pollution on maternal health and indirectly on water security and malnutrition, there is an emerging concern related to fetal exposure to pollutants such as black carbon particles and microplastics (10–13). Pregnant women already face increased physiological and psychosocial demands during pregnancy and are, therefore, more vulnerable to the increased stressors presented by climate change. This was emphasised by the recent WHO Call for Climate Action at the 2023 United Nations Climate Change Conference (COP28), which drew attention to the underestimated and underreported impact of climate events on vulnerable populations, including pregnant women, newborns and children.

This review aims to explore the relationship between climate change and environmental pollution and their impacts on maternal and fetal health, considering both direct and indirect stressors such as extreme weather events, resource insecurity, and exposure to pollutants. We will assess the latest literature, identify knowledge gaps, and suggest strategies to mitigate these effects through focused research and policy interventions. In doing so, this review highlights the urgent need for climate-conscious healthcare policies and stresses the vital role of maternal health within the broader context of climate justice initiatives.

This narrative review was developed as part of a planned meta-analysis on the effects of climate change on adverse pregnancy outcomes and maternal-fetal health. The search strategy included a comprehensive search of peer-reviewed literature and guidelines published between January 2000 and October 2024 using PubMed, Scopus, and Web of Science. Search terms comprised combinations of “climate change,” “pregnancy,” “maternal health,” and related concepts. Original research articles, systematic reviews and meta-analyses in English focusing on climate- and environmental- impacts on maternal-fetal health, were included, while policy documents, including some published in early 2025, were also considered.

The review also summarises the initiatives and position statements of medical association, such as the Royal and the American College of Obstetricians and Gynaecologists (RCOG, UK, and ACOG, US, respectively) to adapt to the effect of climate change and address its specific impacts on women's health, with a focus on healthcare systems in the UK.

## Current evidence on the impact of climate change on adverse pregnancy outcomes

**Figure 1:**
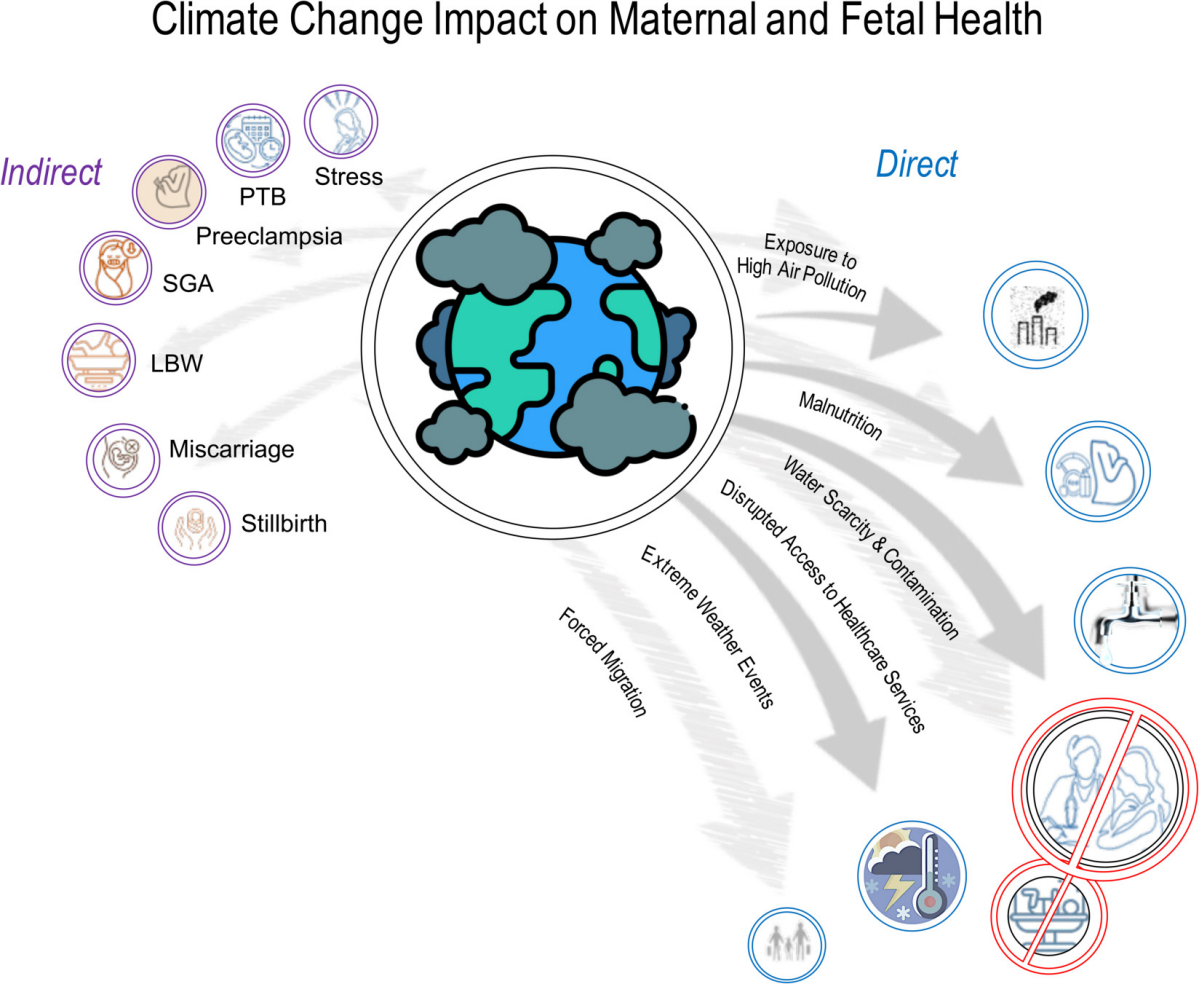
The frequency of climate-related emergencies, such as extreme weather events (heatwaves, floods), natural disasters, and disease outbreaks, is rising. Evidence increasingly links adverse pregnancy outcomes to climate change and maternal exposure to environmental pollutants. Disruptions in healthcare infrastructure after such events can delay or limit care for pregnant women, impacting fetal health. These adverse outcomes may include miscarriage, preterm birth (PTB), stillbirth, fetal growth restriction (FGR), low birth weight (LBW), hypertensive disorders of pregnancy (HDP) like gestational hypertension and pre-eclampsia, gestational diabetes, maternal stress, postpartum depression, and maternal death (5, 18–25, 43, 55, 108–112, 115–117, 119–120, 122–129, 131–136, 138–140, 142–144, 148–153, 155–166, 168, 169, 176–182).

Adverse pregnancy outcomes include a wide range of conditions such as miscarriage, preterm birth (PTB), stillbirth, fetal growth restriction (FGR), or low birth weight (LBW), hypertensive disorders of pregnancy (HDP) including gestational hypertension (GH) and pre-eclampsia (PET), gestational diabetes (GDM), postpartum depression, and maternal death (14–17). Adverse pregnancy outcomes can result from additional factors, including maternal age, pre-existing health conditions, lifestyle factors such as smoking and substance abuse, inadequate prenatal care, deprived socioeconomic status, environmental exposures, and genetic factors. Recently, growing evidence suggests that adverse pregnancy outcomes are also directly and indirectly linked to the consequences of climate change and maternal environmental exposure to pollutants (5, 13, 18–25, 43, 55, 108–112, 116, 116, 119, 120, 127–129, 131–136, 138–140, 142–144, 148–153, 155–160, 162–165, 167, 169, 177, 178, 180–183) (Figure 1).

The changing biosphere and atmosphere threaten food security through droughts, floods, and heatwaves, reducing crop yields and making arable land inhospitable. This can disproportionately impact socioeconomically deprived populations who may lack the resources to adapt to changing conditions, worsening income inequalities and immediate health burden, and affect certain vulnerable groups, e.g., pregnant women (26, 27). Data from the 2019 Global Burden of Disease (GBD) study by the Institute for Health Metrics and Evaluation (IHME) highlight that maternal malnutrition remains a critical concern, especially in low Socio-Demographic Index (SDI) regions and among pregnant adolescents, underlining the need for targeted interventions (28, 121). SDI ranks regions from low to high based on income, education, and fertility rates; regions with low SDI scores often face limited healthcare access, higher disease rates, and lower life expectancy, highlighting areas needing interventions to reduce health disparities (29). Poverty intensifies malnutrition, with pregnancies being especially vulnerable, whereas the WHO stresses the importance of nutrition during the first 1,000 days from conception to age two for lifelong health (30). Women with low BMI face higher risks of FGR whereas maternal iron deficiency significantly increases maternal mortality accounting for at least 20% of deaths, often due to complications (27, 29, 31–36).

Emergency situations, including those caused by extreme weather events (such as heatwaves, floods, droughts, and wildfires), as well as natural disasters (e.g., earthquakes and tsunamis) and disease outbreaks, are increasing in frequency, intensity, and impact, partly due to climate change, and can directly affect maternal and fetal health (WHO, Climate Change and Health). Evidence is still limited but increasing (37), and recognising these challenges, future disaster preparedness efforts must prioritise swift and effective responses to safeguard maternal and fetal health amidst the growing impacts of climate change. Lessons from past disasters (38, 39), such as Hurricane Katrina left approximately 56,000 pregnant women and 75,000 infants directly affected by the devastation (40), and Nepal's earthquakes (41), stress the importance of timely government and humanitarian responses. Disruptions in healthcare infrastructure following natural disasters can lead to delayed or inadequate medical attention for pregnant women and fetal health (WHO, Climate Change and Health), with evidence suggesting increased risks of complications like preterm birth and LBW (42–44). Pregnant women and infants may also be susceptible to metabolic syndrome and impaired neurodevelopment in the aftermath of natural disasters (45–49).

Gestational age at heat exposure has been linked to adverse outcomes from both acute and chronic heat exposure (18, 50). Exposure to extreme heat during pregnancy has also been investigated to assess the risk of stillbirth (51). Heat-exposure is associated with the risk of miscarriage (52) and both low and high temperatures are associated with an increased risk of preterm birth (53). A specific threshold for temperature effects has not been identified, as most studies consider exposure to be temperatures above the 90th percentile for that population (50, 54–58). Proposed mechanisms include increased dehydration and impaired maternal thermoregulation. It remains unclear if heat exposure directly affects thermoregulation in pregnant women or acts as an external stressor triggering hormonal responses (59) as both pathways can impact fetal development and endocrine balance in neonates. Additionally, severe weather events related to climate change can elevate maternal stress levels, potentially leading to complications like preterm birth and LBW (60–63).

Climate change intensifies water security through altered weather patterns, leading to floods, droughts, and polar ice melt, which raises sea levels and salinates fresh water. Notably, access to safe drinking water remains a global issue, with 1 billion people lacking access (64), and a review of 14 studies linked water scarcity and poor sanitation to maternal mortality (65). Industrial pollution further contaminates fresh water, impacting hygiene and safety (66). A 2002 review found moderate evidence of water contaminants causing SGA, neural tube defects, miscarriage, and congenital abnormalities (11). In addition, two US-based studies showed that in New Jersey, contaminated water was linked to small-for-gestational-age (SGA) births and increased incidence of prematurity (67), whereas in Pennsylvania (USA), even low-level water contamination was linked to reduced fertility and adverse birth outcomes, such as low birth weight (68). Significant associations between water contaminants like BPA, phthalates, and PFAS with adverse obstetric outcomes and gynaecological pathology, including higher rates of infertility and endometriosis have also been reported (69).

Furthermore, and despite the rise in global concern around air pollution, global emissions continue to grow, and the problem persists. According to the WHO, household air pollution causes 3.2 million premature deaths annually, with over 99% of people living in areas exceeding WHO air quality standards (70). Ambient air pollution, primarily from motor vehicles, solid fuel burning, and industry, causes 4.2 million deaths per year. Air pollution is made up of a collection of solid and/or liquid materials of various sizes, ranging from a few nanometers in diameter, consisting of particulate matter (PM10 and PM2.5) and gaseous pollutants like sulphur dioxide, nitrogen oxides, ozone, and carbon monoxide (71). Associations between air pollution and pregnancy outcomes are complex due to various pollutants and confounding factors, but there is evidence associating certain types of air pollution and adverse birth outcomes, including, premature delivery, low birthweight, pre-eclampsia and miscarriage (5, 58, 72, 146). Notably, wildfire-specific PM2.5 exposure in the last four gestational weeks was shown to increase risks of preterm birth, stillbirth, and nonvertex presentation, whereas black carbon exposure was strongly linked to stillbirth, and the most vulnerable groups were female births, mothers with low socioeconomic status, and those with high biothermal exposure (18).

**Figure 2:**
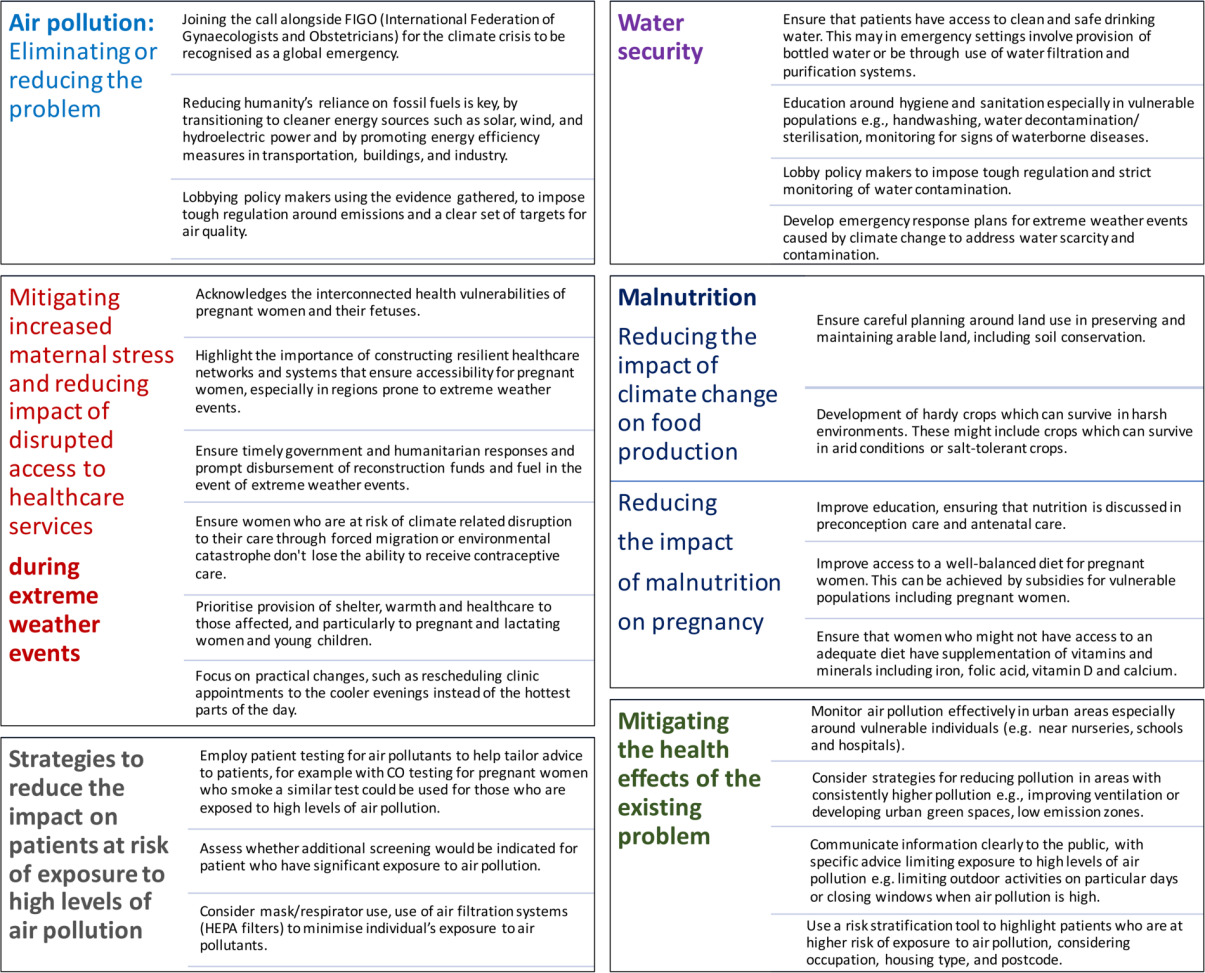
Strategies to mitigate climate change impacts on maternal health: Air pollution, water security, healthcare access, and nutrition.

Raising awareness of climate and environmental change's impact on women's health, including transgenerational effects, is crucial [(73, 161–166, 168–182) and Figure 1]. Researchers and health organizations are examining all these climate change induced challenges, emphasizing the urgent need to protect pregnant women. Transdisciplinary research into the pregnancy exposome is essential to address health disparities, identify at-risk populations, and understand the molecular mechanisms behind climate-induced pregnancy complications (73). Moreover, efforts should focus on the urgency to protect women against the growing threats of a changing climate and environment, and strategies to understand these impacts while developing strategies to reduce, mitigate, or eliminate risks to maternal and fetal health (Figure 2) (4, 14, 40, 80, 113, 114, 118, 137, 141, 147, 154, 170). These efforts should prioritize minimizing the healthcare system's carbon footprint, implementing early interventions to reduce its environmental impact, empowering healthcare professionals to educate patients, and encouraging them to advocate for policy changes.

## Climate action in high-income healthcare systems: lessons from the UK

High-income countries (HICs) bear particular responsibility for climate mitigation given their disproportionate carbon emissions and resource-intensive healthcare systems (74). The United Kingdom has emerged as a global leader in climate-conscious healthcare reform, with the National Health Service (NHS) implementing its landmark Net Zero Strategy in 2020, the world's first national health system commitment to carbon neutrality by 2040 (75). This initiative combines operational changes (e.g., low-carbon anaesthesia) with systemic interventions (e.g., supplier emissions requirements) demonstrating how industrialized nations can combine quality care with sustainability (76, 77). The UK experience also reveals both the promise and the challenges of healthcare decarbonization. While the NHS has reduced emissions by 30% since 2010 through innovations like virtual consultations and green surgical protocols, barriers persist in areas such as pharmaceutical supply chains and medical device lifecycle management (149). These lessons provide actionable insights for other HICs and LMICs facing comparable infrastructure challenges, from hospital energy use patterns to climate-vulnerable patient populations (76, 78). Therefore, the UK NHS model accentuates that healthcare sustainability requires both technological solutions and policy frameworks that can facilitate systemic change (79).

## Reducing the carbon footprints of the healthcare system

The healthcare system should be leading by example in the implementation of carbon-neutral and emission-free provision of care and take responsibility for the supply chain (80). In total, the healthcare sector accounts for between 4%–5% of global carbon emissions (81, 82), thus, implementing sustainable practices in obstetrics can significantly reduce emissions and waste. Additionally, adopting energy-efficient measures, including transitioning hospitals to 100% renewable energy, as advocated and practiced by the RCOG, is crucial for sustainability.

Proper waste segregation and mindful practices, such as reduction of unused medical supplies disposal, can help lower the environmental impact of waste management. A recent systematic review assessed studies on the environmental impact of vaginal births, obstetric and gynaecological surgical procedures, menstrual products, vaginal specula and transportation to gynaecological oncologic consultations (83). The results showed that among the highest yielding mitigation strategies are displacing disposable with reusable materials and minimising content of surgical custom pack, whereas the lowest-yielding mitigation strategy was waste optimisation, including recycling. For caesarean sections, when excluding energy costs and analgesia, over half of the carbon impact comes from disposables, such as single-use drapes (84).

Professional bodies for obstetricians, gynaecologists, and anaesthetists should collaborate to develop standardized guidelines that prioritize women's health while effectively reducing the climate impact of maternity care. When inhalation anaesthesia is used, it can contribute up to 63% of the carbon footprint of perioperative departments in high-income countries, with anaesthetic gas emissions accounting for approximately 2%–5% of the total carbon footprint of the healthcare sector (75, 77, 85). When exploring alternative analgesia options, it is essential to prioritize patient safety and choice without compromising care (86). Additionally, careful selection of anaesthetics is vital, particularly by eliminating gases, such as desflurane, which lack significant clinical benefits but carry higher economic and environmental costs (87). Perhaps for maternity care where caesarean sections are more commonly performed under spinal anaesthesia, labour analgesia presents a more significant opportunity to reduce emissions. Nitrous oxide mixed with oxygen (Entonox) is widely used but has one of the worst carbon footprints. It was shown that for vaginal birth, using morphine resulted in a carbon footprint of 9.48 kg CO2e compared to 246.73 kg CO2e with Entonox (84). Strategies to reduce the impact of Entonox include reducing its use and replacing it with alternative analgesics, recapture or breakdown expired nitrous oxide and decommissioning leaky nitrous oxide manifolds. Notably, a recent study found that postnatal women had low awareness of nitrous oxide's environmental impact (88). However, after receiving information, 99% believed they had the right to be informed about its harmful effects when choosing analgesia.

Surgical interventions in obstetrics, such as caesarean sections, are highly carbon-intensive and generally have a larger carbon footprint than vaginal births when analgesia is excluded. This comparison is dependent on the type of analgesia used, with the use of nitrous oxide with oxygen during vaginal birth increasing its carbon footprint by 25 times (84), highlighting the complexity of assessing environmental impacts in maternity care. In the UK, the surgical Royal Colleges have developed evidence-based interventions to reduce the environmental impact of surgery (UKHACC). The approach accentuates preventative measures to reduce the need for surgery and promotes sustainability through the Intercollegiate Green Theatre Checklist (89). This strategy focuses on reusing equipment and minimising reliance on energy-intensive alternatives (90).

## Reducing environmental impact of healthcare through early interventions and innovation

Preventative medicine is a crucial strategy to reduce the environmental impact of healthcare, as improved screening and preventative measures can help decrease the need for interventions. For example, pelvic floor exercises during and after pregnancy can reduce the rate of incontinence and long-term reliance on single-use continence pads (91). A recent study evaluated patient satisfaction on gynaecological examination with metal, plastic and biobased plastic vaginal specula, and investigated whether patients are willing to compromise on comfort for a more sustainable healthcare system (92). The results indicated a significant difference in favour of a biobased plastic speculum, with patients willing to compromise on comfort in favour of sustainability. While the willingness to use reusable specula is important, expecting women to endure increased discomfort for the sake of sustainability contradicts gender-transformative principles. Women should not be required to bear additional burdens in efforts to “greenify” healthcare systems. Instead of compromising comfort, alternatives such as self-sampling methods, miniature-imaging devices for cervical imaging and sampling, and redesigned specula should be explored. For example, innovative speculum designs that prioritize patient comfort while being compatible with high-level disinfection and autoclaving offer a sustainable, hygienic solution for gynaecological care (93–95).

## Initiatives and calls to action

In the UK, the RCOG is working closely with key partners to develop and implement lower-carbon NHS care models (75, 96). The project actively involves individuals with lived experience of maternity services to ensure equitable and system-wide benefits. Supported by the findings of this project the RCOG seeks to integrate sustainability into care guidelines, including future Green-top Guidelines. As part of this effort, clear guidance should be given to patients on recommended safe, acceptable, and sustainable period products (97, 98).

Additionally, the RCOG, Royal College of Paediatricians and Child Health (RCPCH), and UK Health Alliance on Climate Change (UKHACC) have called on the UK government to create a heatwave plan that specifically addresses the needs of pregnant women and children. This call comes in response to evidence that severe weather events linked to climate change can increase maternal stress levels, potentially leading to complications (RCOG, RCPCH, and UKHACC call). In the US, ACOG recently released a committee statement on disaster preparedness, highlighting the critical needs of obstetric and gynaecologic patients during emergencies (99). Pregnant, postpartum, and breastfeeding women can receive guidance for emergency plans, including evacuation strategies, supply and birth kits, and access to important documents, from resources such as the CDC and the American Red Cross.

## Healthcare professionals as patient educators

There is clear data linking climate change to negative maternal and fetal health and yet there has been limited success in implementing strategies to reduce its impact. When looking at implementing evidence-based practice, Morena et al. found clinical champions were a very successful way to reduce barriers to behavioural change (100). Clinical champions are individuals with great communication skills, committed to driving, advocating for, and supporting the implementation of initiatives while addressing and overcoming potential resistance at the organizational level (101). Green champions can lead initiatives such as waste reduction and energy efficiency, promoting eco-friendly practices in healthcare. They can stimulate a culture of climate-conscious care by educating colleagues both about the health impact of climate change, particularly on pregnant women, and the impact of sustainability on the reduction of carbon footprint. Additionally, they help pregnant women, and their families understand the importance of sustainable practices, encouraging healthier behaviours and environmental responsibility in maternal and fetal healthcare. Perhaps ensuring that maternity and women's health services are empowered with green champions who can spearhead organisational change would be a big step forward.

Healthcare professionals need training in sustainability and climate issues to effectively educate patients. In the UK, the RCOG is expanding sustainability education through training programs, an e-Learning course on climate change and healthcare, and workshops on integrating sustainability into patient discussions (RCOG, eLearning Catalogue). Additionally, the Centre for Sustainable Healthcare offers courses to deepen professionals' understanding of the direct and indirect impacts of climate change (102). As discussions around the mode of delivery and birth plans are highly personal and must be approached sensitively, decision aids can be valuable tools in guiding patients (103). Decision aids that incorporate clinical evidence alongside sustainability considerations can help inform patient choices, promoting environmentally conscious healthcare while maintaining high-quality, women-centered care.

## Healthcare professionals raising awareness among policy makers

**Figure 3:**
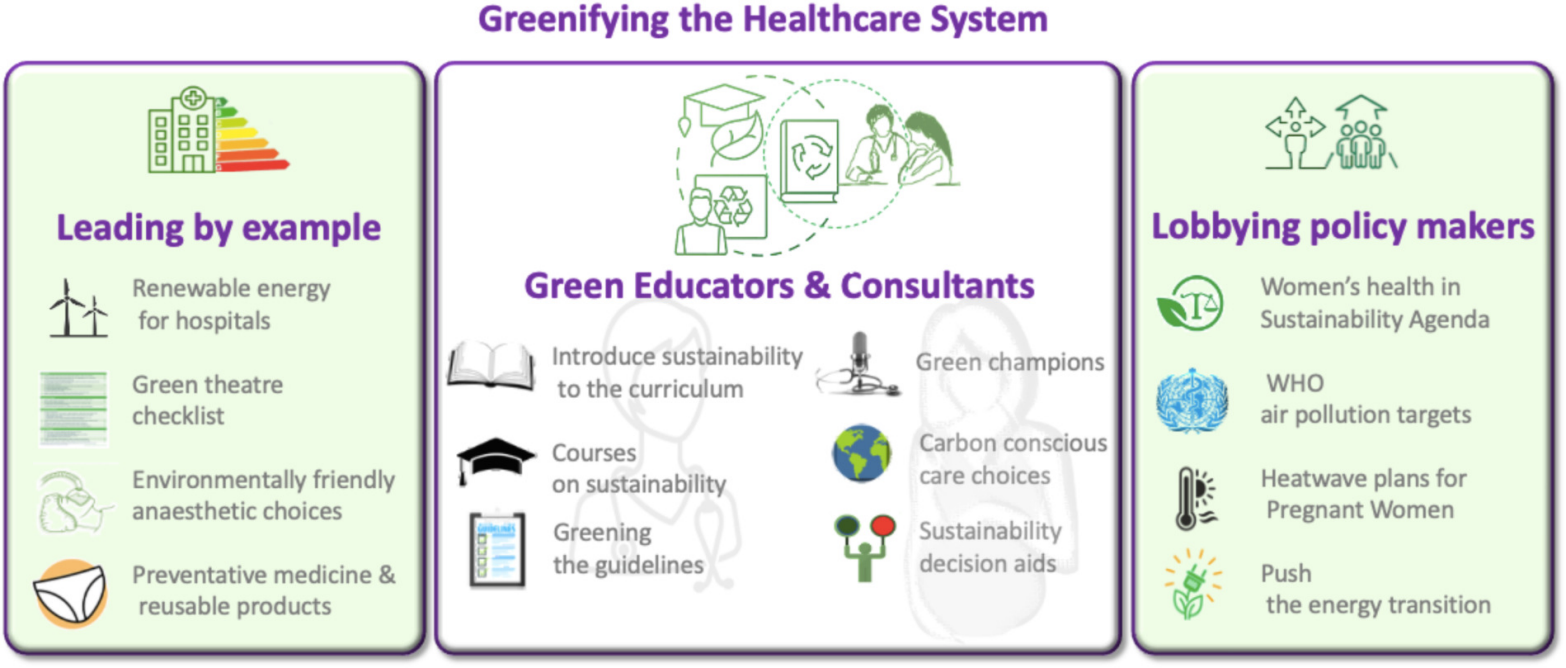
Greenifying the healthcare system infographic.

Despite growing efforts by health professionals to address climate-related health risks, evidence on the effectiveness of interventions is lacking and supporting literature remains scarce (104). Healthcare professionals have a duty to highlight health concerns, due to climate change and environmental pollution, in particular to policy makers (Figure 3). Medical colleges often act to coordinate and advocate for their patients when lobbying the government. In the UK, the RCOG, RCPCH, and UKHACC have jointly identified three urgent actions to protect child, fetal, and maternal health, as outlined in the *Lancet Countdown* policy brief (105). These include enforcing the ambitious air quality standards set by the WHO, developing heatwave response plans with a focus on vulnerable populations, and accelerating the energy transition by cutting fossil fuel subsidies and redirecting funds toward renewable energy. Similarly, the American College of Obstetricians and Gynaecologists (ACOG) has emphasized in a position statement the urgent need for clinical and community-based research on the health impacts of climate change, particularly on women and marginalized populations (106). AJOG also advocates for policies that reduce greenhouse gas emissions, promote environmentally responsible medical practices, and mitigate climate-related health risks, urging national and international leaders to take action and invest in solutions that protect women's health and well-being.

Further to these main policy goals, there are some more specific strategies to reduce the impact of climate change and environmental pollution on maternal and fetal health (Figure 2). These involve wider policies to reduce or prevent ongoing pollution, and solutions to mitigate the effects of air pollution, polluted water, poor diet, and extreme weather events on pregnant women and their pregnancies.

## Discussion

The climate crisis presents significant risks to women, especially maternal and fetal health (Figure 1), with environmental exposure to pollutants disproportionately impacting women facing socio-economic hardships. Therefore, a woman-centered approach to healthcare is essential, focusing on inclusive, personalized care that addresses the unique physiological, emotional, cultural, and socio-economic needs of pregnant women. This approach is decisive as maternal health directly affects both women's health and the development of their pregnancies. Therefore, climate crisis-centered guidelines and policies should prioritize women's health across their lifespan, with a particular emphasis on maternal and fetal health during pregnancy and beyond.

While individual efforts to protect pregnant women are important, climate change requires global strategies and solutions. Raising public awareness and advocating for policy reforms are vital to create meaningful change on a larger scale. Mental health support, management of chronic conditions, such as gestational diabetes and hypertension, and health equity must also be prioritized to ensure all women have access to quality care, regardless of their background. In addition, integrating sexual and reproductive health and human rights into climate policies is critical to enhance the well-being and health of women globally (2, 130).

Furthermore, advanced guidelines that incorporate AI-driven solutions, such as predictive algorithms for risk assessment and personalized healthcare recommendations, can significantly augment the precision of women's health and prenatal care. Thus, responsible AI deployment, aligned with climate justice principles, can help minimize environmental impact while improving healthcare delivery (107).

Moreover, healthcare systems can lead by adopting greener practices, engaging green champions, educators, and policymakers to prioritize sustainability. This includes positioning women's health at the forefront of the sustainability agenda, educating healthcare professionals about sustainable practices and greenifying healthcare operations (Figure 3). These initiatives will not only help mitigate climate crisis-related health risks but also ensure equitable healthcare access for these vulnerable groups, supporting the broader goal of climate justice.

Women's healthcare providers play a crucial role in educating patients about the adverse health impacts of climate change and advocating for sustainable practices. As some of the most trusted voices on climate issues, healthcare providers must lead by example, implementing climate change mitigation guidelines within clinical settings. By combining education with actionable interventions, they can empower patients and contribute to broader efforts to combat climate change's impact on public health.

While educating patients about climate change is important, true systemic change requires broader action beyond individual behaviour. OB-GYNs can amplify their roles through education, research, and advocacy (4), but we must also recognize the need for institutional and policy-level shifts. In line with this, the FIGO Opinion outlines four key recommendations for policymakers and stakeholders to reduce and prevent exposure to toxic chemicals globally, i.e., to reduce and prevent exposure to toxic chemicals: advocating for preventive policies, ensuring a healthy food system, integrating environmental health into healthcare, and promoting environmental justice (FIGO).

Balancing these systemic changes with patient education is essential for lasting impact. Recognising the climate crisis as a global emergency, researchers and healthcare providers should lead advocacy, research, capacity building, and education efforts to address its health consequences.
